# Case report: Nasopharyngeal tuberculosis

**DOI:** 10.4103/0971-3026.38507

**Published:** 2008-02

**Authors:** BKD Prasad, GS Kejriwal, SN Sahu

**Affiliations:** Maharajah's Institute of Medical Sciences, Nellimarla, Vizianagaram, India

**Keywords:** Nasopharynx, tuberculosis

Tuberculosis is a common infectious disease worldwide, including India. It most commonly affects the lungs, though any organ can be affected. Upper respiratory tract involvement is uncommon (1.8%) and involvement of the nasopharynx (0.1%) is rarer still.[1] Tuberculous involvement of the nasopharynx may be primary, without involvement of any other system, or secondary to pulmonary or extrapulmonary involvement. There is limited description of this entity in the classic otolaryngology textbooks.[2,3] We would like to describe a case of primary tuberculous involvement of the nasopharynx.

## Case Report

**Figure 1:**
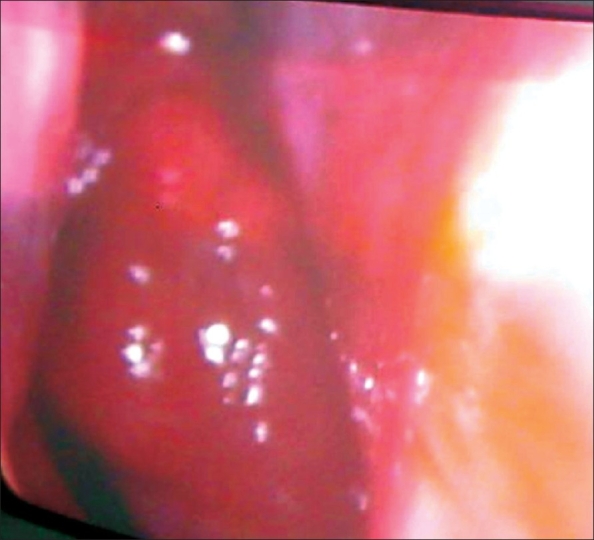
Nasal endoscopy image shows a glistening, red mass

A 22-year-old man came with a history of nasal obstruction, mouth breathing, and snoring for 3 years. He had no past history of nasal bleeding or any other throat or ear complaints. Clinical examination showed nothing significant. The ESR was elevated to 110 mm/1 h. The Mantoux test showed an induration of 24 mm. The chest radiograph was normal. Nasopharyngeal endoscopy showed a pink, glistening mass involving the whole of the nasopharynx, occluding both choanae [Figure 1].

**Figure 2:**
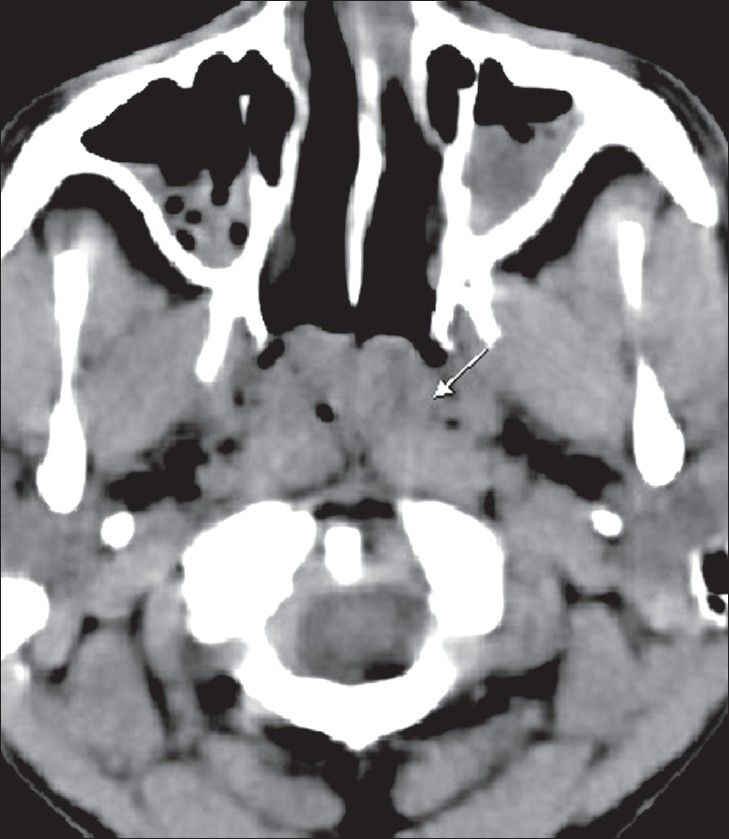
Plain CT scan image shows a soft tissue mass lesion in the roof of the nasopharynx (arrow)

**Figure 3 (A-C):**
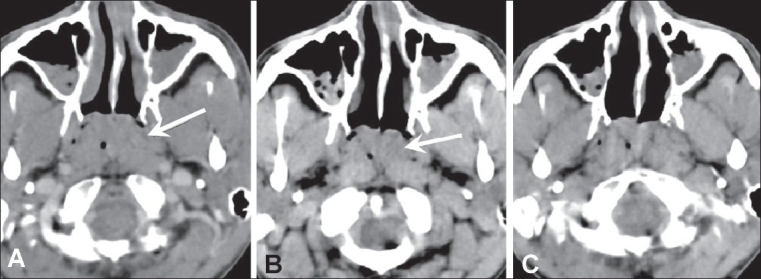
Contrast-enhanced CT scan images shows a moderately enhancing mass (arrow) extending up to the posterior choanae

CT scan showed a moderately enhancing mass, measuring 4.0 × 2.7 × 4.0 cm, in the nasopharyngeal roof, extending up to the posterior choanae bilaterally [Figures 2 and 3]. The surrounding fat planes were well-maintained, without involvement of adjacent structures. The bones were not involved and there was no cervical lymphadenopathy. An endoscopic biopsy showed multiple granulomata, caseous necrosis, Langerhans giant cells, and epitheloid cells. Tissue PCR was positive for tuberculosis.

## Discussion

Isolated nasopharyngeal tuberculosis is a rare condition even in endemic areas.[4] Primary nasopharyngeal involvement probably occurs due to reactivation of dormant acid fast bacilli in the adenoids or due to direct mucosal infection after inhalation of the bacilli.[5] It may be commoner than secondary involvement,[6] which usually occurs in conjunction with pulmonary tuberculosis.

The clinical presentation may vary. The patient may be completely healthy, with no underlying disease and with no history of contact with tuberculosis, or may present with a sore throat.[7] Other presentations include epistaxis, running nose, postnasal drip, nasal obstruction, and chronic cough. Cervical lymphadenopathy is a common accompaniment,[6] followed by nasal discharge and obstruction. Tuberculous involvement of the nasopharynx may be underdiagnosed, since it does not produce obvious symptoms and physical signs in all cases.[6] Atypical presentations with diplopia[5] and snoring[4] have also been reported.

Endoscopic examination may reveal a polypoidal mass, ulceration, plaque, or diffuse mucosal thickening.[8–10] All these findings may suggest nasopharyngeal carcinoma, lymphoma, or Wegener's granulomatosis.[11] Infections such as syphilis, leprosy, and fungal diseases may have a similar appearance. Tuberculosis may coexist with malignancy[12] and has even been described after radiotherapy.[13]

Plain radiographs are usually not useful and may show a nonspecific soft tissue lesion in the nasopharynx or may simulate adenoid hypertrophy in young patients. CT scan commonly shows either diffuse mucosal thickening or a moderately enhancing polypoidal mass in the roof of the nasopharynx, which may be ulcerated.[13] Necrosis may be seen. MRI commonly shows a mass or diffuse mucosal thickening of intermediate signal intensity on T1W and T2W sequences, with moderate contrast enhancement on T1W images.[8]

It is difficult to make an accurate diagnosis of nasopharyngeal tuberculosis on imaging findings alone and a biopsy is required to confirm the diagnosis and to differentiate it from malignancy and the other conditions described above.

Since our patient did not have lung involvement or lymphadenopathy, it is likely that this was a case of primary nasopharyngeal tuberculosis.
